# Relationship between lean body mass, limb asymmetry, and eccentric hamstring strength in NCAA division I football players

**DOI:** 10.1080/15502783.2025.2550200

**Published:** 2025-08-30

**Authors:** Emma E. Worley, Gianna F. Mastrofini, Sten O. Stray-Gundersen, Alexander A. Andersen, Shawn M. Arent

**Affiliations:** University of South Carolina, Arnold School of Public Health, Department of Exercise Science, Columbia, SC, USA

**Keywords:** Body composition, injury risk, Athletes, Muscular imbalance

## Abstract

**Background:**

Eccentric hamstring strength and limb asymmetry are important factors in performance and injury risk among American football players. Given that lean mass (LM) contributes to strength and power development, examining its relationship with eccentric hamstring strength and limb asymmetry may provide valuable insights for enhancing performance and reducing injury risk. Therefore, the purpose of this study was to determine how whole-body LM, lower body LM, and lower body LM asymmetry relate to eccentric hamstring strength and strength asymmetry in collegiate football players.

**Methods:**

Fifteen National Collegiate Athletic Association (NCAA) Division I football players (age = 18.5 ± 1.1y, height = 185.7 ± 4.1 cm, weight = 88.3 ± 8.8 kg) underwent body composition analysis and eccentric hamstring strength assessment (NordBord, Vald Performance, Version 1.0) following an eight-week winter off-season period. Dual-energy x-ray absorptiometry (DEXA) was used to assess whole-body LM, lower body LM, and lower body LM asymmetry index between the dominant and non-dominant legs. The best of three repetitions of the Nordic hamstring test were recorded to assess maximal eccentric hamstring force production. Lower body LM and eccentric hamstring force production asymmetries were calculated by subtracting non-dominant leg values from dominant leg values. Pearson-product moment correlations were used to quantify relationships between each variable (α = 0.05).

**Results:**

Non-dominant leg eccentric hamstring force production negatively correlated with eccentric hamstring force production asymmetry (*r* = −0.65, *p* = 0.01). Lower body LM was associated with bilateral eccentric hamstring force production (*r* = 0.78, *p* < 0.01). No significant correlations were observed between whole-body LM and bilateral eccentric hamstring force production (*p* > 0.05). Additionally, lower body LM asymmetry did not correlate with eccentric hamstring force production asymmetry (*p* > 0.05).

**Conclusion:**

Greater non-dominant eccentric hamstring strength was associated with greater bilateral hamstring strength symmetry. In addition, greater lower body LM strongly correlated with bilateral eccentric hamstring strength. The lack of correlation between lower body LM asymmetry and eccentric hamstring strength asymmetry suggests that factors beyond lower body LM contribute to functional imbalances. These findings highlight the impact of non-dominant leg strength training in promoting bilateral hamstring strength symmetry in collegiate football players. Incorporating single-leg exercises to reduce the gap between non-dominant and dominant leg strength may help reduce limb asymmetry, potentially improving performance and lowering injury risk. Future research should include positional subgroup analyses to ascertain whether positional demands influence these relationships and help practitioners address common functional imbalances in collegiate football players.

